# Comparison of outcomes between intracapsular resection and pseudocapsule-based extracapsular resection for pituitary adenoma: a systematic review and meta-analysis

**DOI:** 10.1186/s12883-022-02574-9

**Published:** 2022-02-12

**Authors:** Xiang Zhang, Yan-Gang Wang, Jiahe Tan, Guanjian Zhao, Mincai Ma, Jin Chen, Ning Huang

**Affiliations:** 1 Department of Neurosurgery, The Second Affiliated Hospital of Chongqing Medical University, Chongqing, 400010 China; 2 Department of Neurosurgery, The First Affiliated Hospital of Chongqing Medical University, 1 Medical Rd, Chongqing, 400016 China

**Keywords:** Pituitary adenoma, Pseudocapsule, Intracapsular resection, Extracapsular resection

## Abstract

**Background:**

Transsphenoidal surgery is the preferred first-line therapy for most pituitary adenoma(PA), and the conventional strategy of treatment is intracapsular resection(IR). The protocol of extracapsular resection(ER), which considers the pseudocapsule as the PA boundary for surgical removal, has also been introduced gradually. In this study, the clinical efficacies and complications were explored and compared between these two procedures.

**Methods:**

A systematic literature review was performed in the PubMed, EMBASE, Web of Science and Cochrane databases. Articles comparing between IR and ER were included.

**Results:**

There were 7 studies containing 1768 cases in accordance with the inclusion criteria. Although the meta-analysis showed no significant difference in complete resection, a sensitivity analysis revealed that ER was more conducive to total PA resection than IR. Moreover, we found a significant difference in favor of ER regarding biochemical remission. Furthermore, there was no significant difference in the incidence rate of certain complications, such as hormone deficiency, diabetes insipidus, intraoperative cerebrospinal fluid(CSF) and postoperative CSF leakage. However, a sensitivity analysis suggested that IR decreased the risk of intraoperative CSF leakage.

**Conclusions:**

This meta-analysis unveiled that ER contributed to biochemical remission. To some extent, our results also showed that ER played a positive role in complete resection, but that IR reduced the incidence of intraoperative CSF leakage. However, the available evidence needs to be further authenticated using well-designed prospective, multicenter, randomized controlled clinical trials.

## Background

Pituitary adenoma(PA) is a common benign neoplasm with a morbidity of 115/100,000 that comprises 10 ~ 15% of primary tumors in the brain [1]. Both compression of surrounding structures and endocrine dysfunction originating from PA are detrimental to quality of life [2]. With the progression of instruments and technologies for microneurosurgery, transsphenoidal surgical resection remains the cornerstone of therapy for most PA, including some cases of prolactin PA [3].

In traditional endonasal transsphenoidal surgery, the PA mass can be removed in an intracapsular fashion similar to internal decompression after opening the endocranium of the sellar floor, but the visual blind zones, dropping of residual tumor and expansion of the normal gland frequently result in failure of complete resection. In addition, the levels of hormones are not able to drop to normal levels for functional PA after the operation [4, 5]. Thus, novel modifications of this procedure have been explored. Increasing evidence has indicated the presence of histologic pseudocapsules around the PA, which contribute to boundary recognition, gross-total excision and endocrinological remission [6]. Therefore, pseudocapsule-based extracapsular resection(ER) is expected to be adopted as a surgical tactic for more radical excision of PA [7]. As a result, the transsphenoidal approach has been categorized into intracapsular resection(IR) and ER. Recently, some articles have focused on the direct comparison of outcomes between the two surgical techniques [5–11]. However, the conflicting results have given rise to arguments that ER could be a source of injury to normal pituitary tissue and increased risks of complications [12]. In fact, it is not clear whether ER shows improved effectiveness and safety compared with IR.

We realized that there was no meta-analysis to confirm the pros and cons of the two surgical methods. Therefore, to clarify this issue, we conducted a meta-analysis in this study.

## Methods

The present systematic review and meta-analysis were performed in accordance with Preferred Reporting Items for Systematic Reviews and Meta-Analysis(PRISMA) guidelines [13].

### Literature search

A comprehensive literature search in the PubMed, EMBASE, Web of Science and Cochrane databases was administered to estimate outcomes between transsphenoidal IR and ER. Search terms included (pituitary adenoma), pseudocapsule, (extracapsular resection), (intracapsular resection) as Medical Subject Headings(MeSH) and their entry terms. The literature search period ended at Aug 9, 2021.

### Inclusion and exclusion criteria

Articles were included according to the following criteria: (1) Population: patients underwent transsphenoidal microsurgery, and pituitary adenomas were identified according to medical record files or pathological diagnosis. (2) Interventions: The pseudocapsule was used as a boundary to distinguish PA from normal structures, and both tumors and pseudocapsules were resected. (3) Comparisons: the pseudocapsule was not removed or no pseudocapsule was observed during operation. (4) Outcomes: Studies showed data regarding complete resection, biochemical remission, hormone deficiency, diabetes insipidus, intraoperative CSF leakage or postoperative CSF leakage. Complete resection was identified as no visible tumor according to intraoperative detection and postoperative imaging. Biochemical remission and hormone deficiency were investigated depending on preoperative, postoperative and follow-up endocrinological examinations, and the hormone follow-up was not less than two months. Biochemical remission was defined by corresponding consensus from their respective countries. Hormone deficiency was considered as new development of postoperative hypopituitarism and aggravation of preoperatively existing hypopituitarism. Diabetes insipidus, intraoperative cerebrospinal fluid (CSF) leakage and postoperative CSF leakage were assessed depending on medical records. The exclusion criteria were as follows: (1) Repetitive articles or cases were excluded. (2) The selective priority was cohort studies and randomized controlled trials (RCTs), and other studies were excluded. Then, the title and abstract were reviewed and full-texts were checked to determine the selected studies.

### Data abstraction

Two reviewers(Zhang and Wang) independently extracted information from each eligible article using a standardized form including the author, publication year, country, research institution, type, sample size, follow-up time, gender distribution, age and outcomes. Any disagreements were resolved by discussion between the two investigators. When necessary, a third reviewer(Huang) helped to reach a consensus.

### Assessment of quality

Two researchers(Zhang and Tan) independently estimated the quality of the 7 cohort studies according to the Newcastle–Ottawa Scale (NOS), which was manifested as a nine-point scale [14]. The scores were 4 for selection quality, 2 for comparability and 3 for quality of outcome. The studies’ quality was categorized as low (0–3 points), moderate (4–6 points), and high (7–9 points). Any disagreements were resolved by consensus between the two investigators.

### Statistical analysis

Review Manager Version 5.3.5 software was used for data analysis, and the risk ratio(RR) with a 95% confidence interval (CI) for these dichotomous variables was calculated. We used the Mantel–Haenszel method to determine the weighted summary RR. Significant RR heterogeneity was tested on the basis of the I-squared (I^2^) statistic. The fixed-effects model was used if I^2^ was less than 50%; otherwise, the random-effects model was preferred. The Sensitivity analysis was used to survey the sources of heterogeneity in which one article was deleted and the rest were analyzed to determine whether the heterogeneity could be eliminated by a single study. *P * < 0.05 was considered statistically significant for outcomes.

## Results

### Literature search

**Fig. 1:**
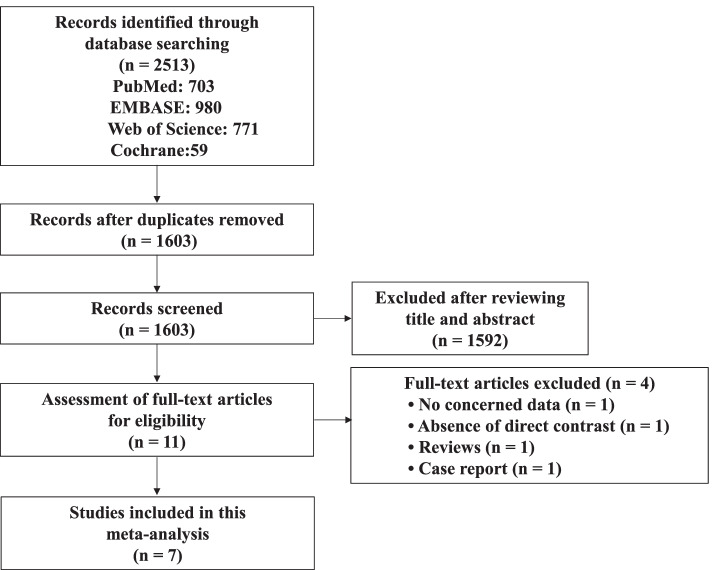
Flowchart for literature searching

The entire literature search process was shown in Fig. 1. The 2531 records were displayed after a comprehensive literature search in the PubMed, EMBASE, Web of Science and Cochrane databases. A total of 1603 records remained for reviewing the title and abstract after deleting duplicate records. Next, 11 articles were selected for full-text evaluation. One without relevant data was eliminated, one was deleted owing to absence of a direct contrast, one was a type of review, and one was a case report. Accordingly, 7 articles were ultimately included in our study, all of which were cohort studies.

### Characteristics of the included studies

The characteristics of the 7 included articles are shown in Table 1. These articles were published from 2005 to 2019, and were carried out by reliable medicine research institutions in 4 different countries. These studies consisted of 1768 cases. The patients’ gender distribution and age in these studies were clear except for the study by Taylor, et al. There were 5 articles with regard to complete resection, biochemical remission, hormone deficiency, diabetes insipidus and intraoperative CSF leakage, respectively. In addition, 4 articles had data on postoperative CSF leakage. The IR and ER protocols were legible in these papers. IR indicated subcapsular resection without removing the pseudocapsule or no pseudocapsule was observed; ER indicated that the pseudocapsule was a boundary for excising the tumor together with the pseudocapsule.

**Table 1:** Summary of characteristics of the included studies

Study (year)	Country	Research institution	Type	Follow up(m)	Sample size	Gender male/female	Age(y)	Outcomes					
								**Complete resection**	**Biochemical remission**	**Hormone deficiency**	**Diabetes insipidus**	**Intraoperative CSF leakage**	**Postoperative CSF leakage**
Li (2019) [5]	China	A hospital affiliated with Anhui Medical University	Cohort study	Median 21	206	IR 28/62 ER37/79	IR Mean 40.2 ER Mean 37.8	IR 62/90 ER 97/116	IR 53/90 ER 89/116	Not clear	IR 57/90 ER 85/116	IR 12/90 ER 31/116	Not clear
Taylor (2018) [6]	USA	A Health System from University of Virginia	Cohort study	2	108	Not clear	Not clear	IR 15/34 ER 49/74	Not clear	IR 7/28 ER 8/69	Not clear	IR 13/34 ER 16/74	Not clear
Kim (2015) [7]	Korea	A hospital and research institute associated with Yonsei University College of Medicine	Cohort study	Mean 57.6	1089	464/625	Mean 43.4	IR 773/826 ER 258/263	IR 302/354 ER 129/143	IR 82/723 ER 36/235	Not clear	IR 344/826 ER 156/263	IR 22/826 ER 11/263
Kawamata (2005) [8]	Japan	A Neurological Institute associated with Tokyo Women’s Medical University	Cohort study	IR Mean 25.2 ER Mean 38.0	48	IR 8/10 ER11/19	IR Mean 44.9 ER Mean 49.8	Not clear	IR 11/18 ER 27/30	IR 1/18 ER 2/30	IR 0/18 ER 0/30	Not clear	Not clear
Kinoshita (2016) [9]	Japan	A hospital associated with Hiroshima University	Cohort study	3	132	IR 33/34 ER33/32	IR Median 61 ER Median 65	Not clear	Not clear	Not clear	IR 10/67 ER 15/65	IR 16/67 ER 26/65	IR 1/67 ER 0/65
Qu (2011) [10]	China	A third grade class-A hospital affiliated with Shandong University	Cohort study	Median 39	142	IR 30/34 ER35/43	Mean 37 ± 1.2	IR 44/64 ER 71/78	IR 45/64 ER 72/78	IR 4/64 ER 8/78	IR 16/64 ER 21/78	Not clear	IR 2/64 ER 6/78
Xie (2016) [11]	China	A hospital and Medical Research Center affiliated with Fudan University	Cohort study	12	43	IR 9/13 ER11/10	IR 47.86 ± 11.62 ER 49.19 ± 12.39	IR 12/22 ER 18/21	IR 12/22 ER 18/21	IR 3/22 ER 1/21	IR 6/22 ER 3/21	IR 8/22 ER 8/21	IR 2/22 ER 4/21

### Data analysis

**Fig. 2:**
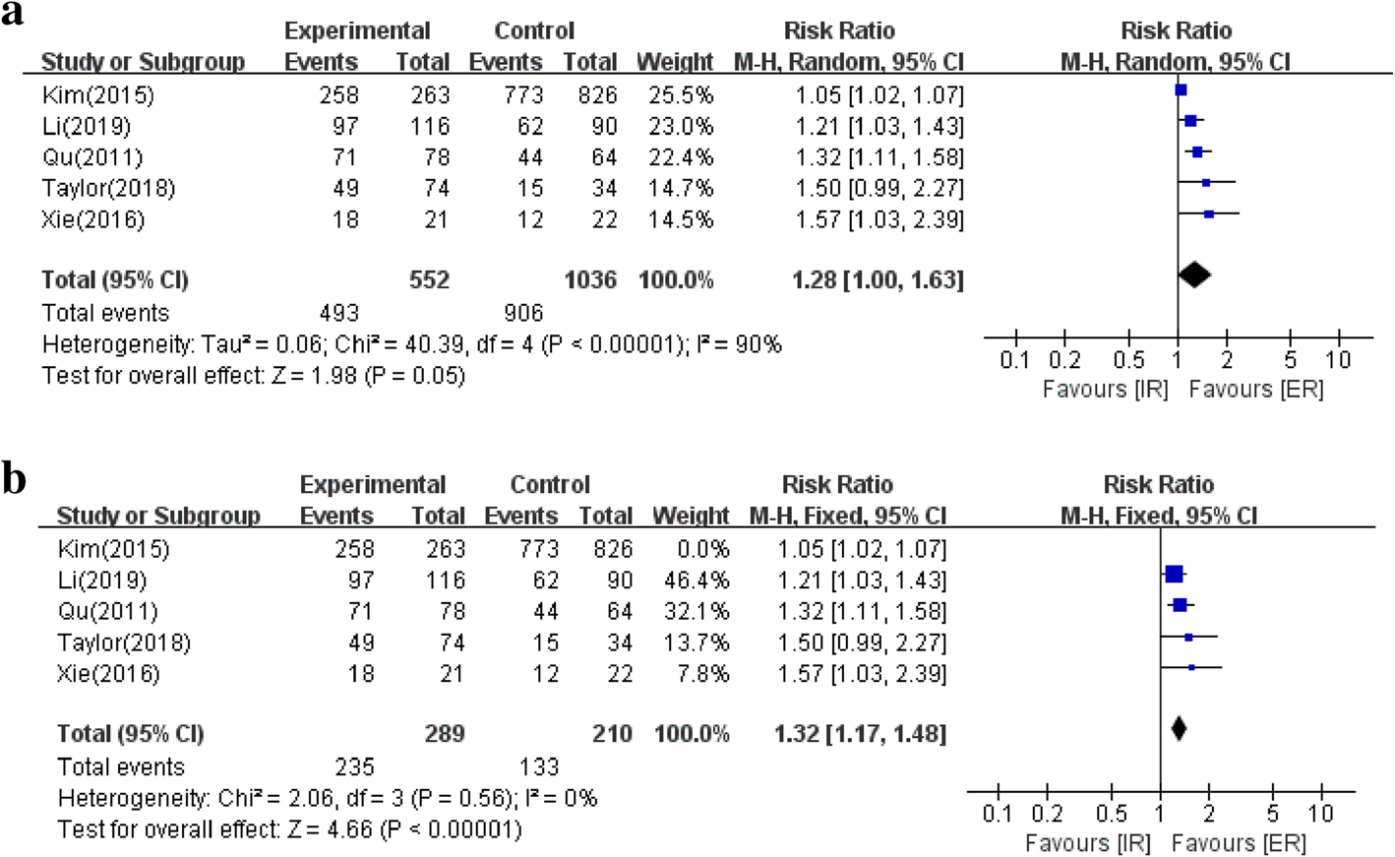
Forest plot to investigate the complete resection in IR and ER groups. **a**. The 5 studies were analyzed. **b**. Sensitivity analysis was performed

As illustrated in Fig. 2a, the 5 studies provided data on complete resection which occurred in 906/1036 (87.45%) cases in the IR group and 493/552(89.31%) cases in the ER group. Pooled analysis found that there was no significant difference between the two groups(RR 1.28; 95% CI 1.00–1.63; *P * = 0.05). Heterogeneity was statistically significant (I^2^ = 90%, *P * < 0.00001), and the source of heterogeneity was examined by a sensitivity analysis (Fig. 2b). When one study was deleted, the heterogeneity(I^2^ = 0%; *P * = 0.56) was decreased and a statistically significant difference was manifested in favor of ER(RR 1.30; 95% CI 1.16–1.45; *P * < 0.00001).

**Fig. 3:**
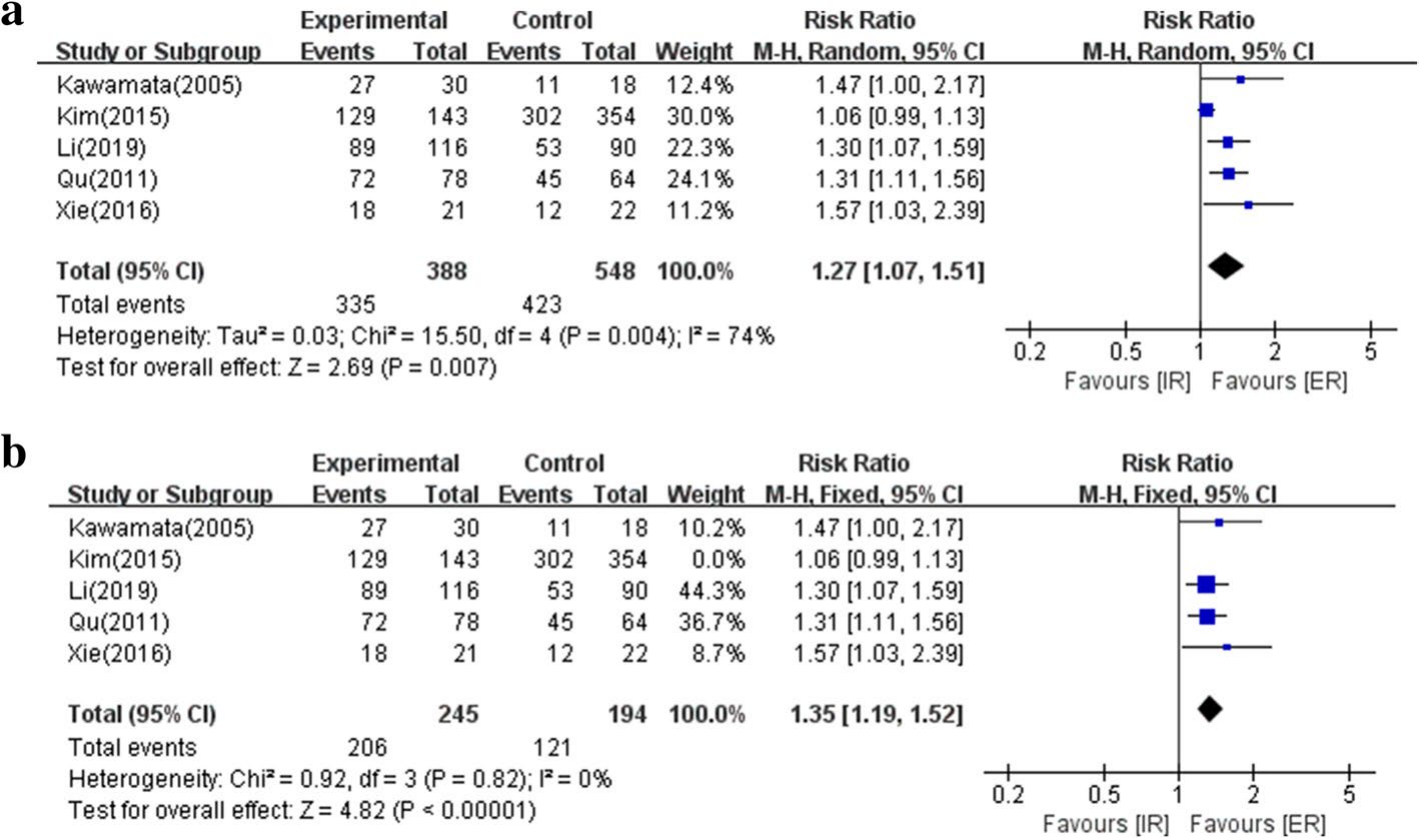
Forest plot analyzing the biochemical remission in IR and ER groups. **a**. The 5 studies were evaluated. **b**. Sensitivity analysis was detected

As shown in Fig. 3a, 5 studies were involved in biochemical remission(423/548, 77.19% in IR; 335/388, 86.34% in ER). Pooled analysis confirmed a statistically significant difference in favor of ER(RR 1.27; 95% CI 1.07–1.51; *P * = 0.007). However, heterogeneity was recognized(I^2^ = 74%, *P * < 0.004), so we performed a sensitivity analysis (Fig. 3b). The results uncovered that ER maintained superiority in biochemical remission(RR 1.35; 95% CI 1.19–1.52; *P * < 0.00001).

**Fig. 4:**
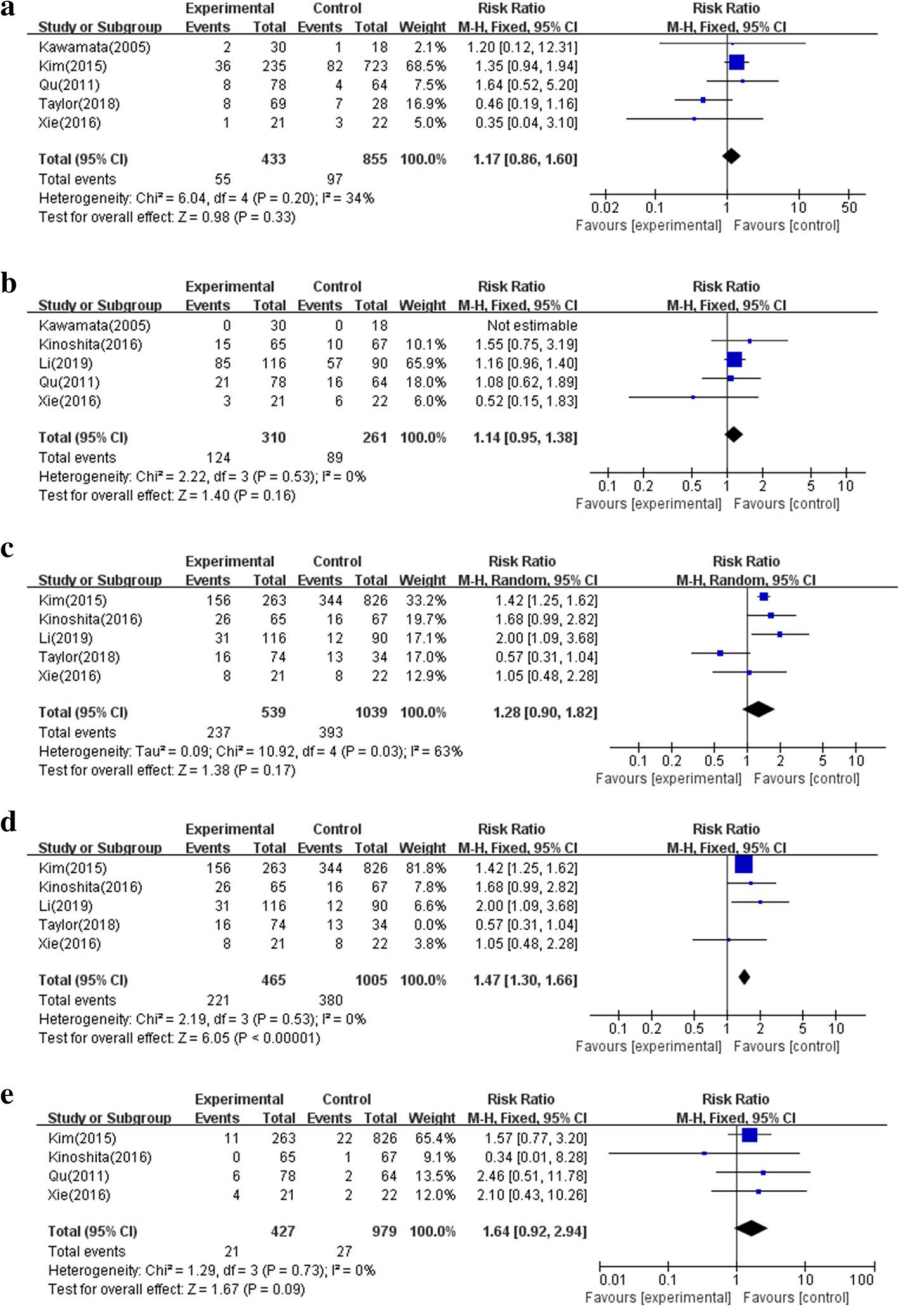
Forest plot to explore the complications between IR and ER groups. **a**. Hormone deficiency. **b**. Diabetes insipidus. **c**. Intraoperative CSF leakage. **d**. Sensitivity analysis of intraoperative CSF leakage. **e**. Postoperative CSF leakage

Next, the complications between the two groups were surveyed. Hormone deficiency, diabetes insipidus, intraoperative CSF leakage and postoperative CSF leakage were reported in 5, 5, 5 and 4 articles, respectively (Table 1). However, no significant difference was proven between the IR and ER groups (Fig. 4a, b, c, e). Heterogeneity emerged with respect to intraoperative CSF leakage(I^2^ = 63%, *P * = 0.03). As a result, a sensitivity analysis was conducted to demonstrate that IR decreased the risk of intraoperative CSF leakage compared with ER (Fig. 4d).

In fact, two papers also described postoperative recurrence. Kim [7] showed that the recurrence was 1.3% (10/773) in IR and 3.1% (8/258) in ER after a mean follow-up of 4.8 years. Qu [10] reported that the relapse rate was 6.8%(3/44) in IR and 0% (0/71) in ER after a median follow-up of 39 months. Qu [10] was the only author to report that the incidence of postoperative visual deterioration was 1.56%(1/64) in IR and 2.56%(2/78) in ER. However, these complications were not analyzed due to deficient literature and very few data.

### Quality of literatures

As shown in Table 2, All 7 cohort studies were high quality (NOS 7–8 points). The approaches for analysis of publication bias, such as funnel plots, would be short of efficacy owing to the small number of articles in this study. Consequently, publication bias was not estimated.

**Table 2:** Results of quality assessment using the Newcastle–Ottawa Scale(NOS) for cohort studies

	**Selection**				**Comparability**	**Outcome**			
**Study**	**Representativeness of exposed cohort**	**Selection of non-exposed cohort**	**Ascertainment of exposure**	**Demonstration that outcome of interest was not present at start of study**	**Comparability of Cohorts on the basis of the design or analysis**	**Assessment of outcome**	**Follow-up long enough for outcomes**	**Adequacy of follow up**	**Quality** **score**
Kawamata (2005) [8]	★	★	★	★	★	★	★	★	8
Kim (2015) [7]	★	★	★	★	★	★	×	★	7
Kinoshita (2016) [9]	★	★	★	×	★	★	★	★	7
Qu (2011) [10]	★	★	★	×	★	★	★	★	7
Taylor (2018) [6]	★	★	★	★	★	★	×	★	7
Xie (2016) [11]	★	★	★	×	★★	★	★	★	8
Li (2019) [5]	★	★	★	★	★	★	★	★	8

## Discussion

Many pituitary adenomas are encased in a compressed, thin layer of normal pituitary tissues, which are defined as pseudocapsules [4]. The basement membrane, capillaries, reticulin envelope, collagen, fibroblasts and pericytes are the histological components of pseudocapsule [8].In recent years, an increasing number of neurosurgeons have paid close attention to this small and delicate structure and they advocate that the PA and its pseudocapsule can be removed together because of the prominent neoplasm boundary for PA [5]. Additionally, it was possible that sparse tumor cells could invade and infiltrate the pseudocapsule, resulting in PA relapse [15].

Several articles confirmed that pseudocapsules helped to identify PA from normal structures, and ER was extremely useful for PA resection, whereas it was necessary to debate whether ER also has disadvantages compared with IR [16]. To the best of our knowledge, no meta-analysis has previously been reported to expose the different outcomes between IR and ER during transsphenoidal surgery for PA. The aim of our meta-analysis was to understand which strategy was conducive to complete resection and biochemical remission as well as reduced risk of complications.

The pooled results revealed that ER was superior to IR for endocrine remission. It was possible that the functional tumor cells hidden in the pseudocapsule would be excised. Unexpectedly, the data pointed out that ER did not cause complete resection. However, a sensitivity analysis was performed in consideration of heterogeneity, which proposed that ER enhanced reliable total removal of inner tumor contents after Kim’s paper was deleted. The reasons involved in the different results were investigated carefully. First, the large samples in Kim’s study provided advantages for clinical research, but the final data extracted from these 5 studies could have been affected by and biased toward Kim’s results which showed a miniscule gap of complete resection between IR(93.6%) and ER(98.1%). Second, although Hardy type IV tumors were excluded by the authors, there was no description about the consistency of tumor volumes and invasive grades in the two groups, which could also lead to operative difficulty and result bias.

The pseudocapsule was regarded as the border between the PA and the normal pituitary gland so that ER avoided erroneous resection of the pituitary gland, whereas additional removal of the pseudocapsule also increased the risk of damage to pituitary function, generating hypopituitarism and diabetes insipidus [17]. CSF leakage is a common complication due to arachnoid injury during transsphenoidal surgery. ER required more aggressive resection of the pseudocapsule which was attached directly to the surface of arachnoid. As a result, the extra process to remove the pseudocapsule from the thin layer of arachnoid enhanced tension on the arachnoid, inducing tearing of arachnoid and CSF leakage [7, 18]. In contrast, some studies have suggested that the advantages of ER for anatomical orientation, and removal of a security margin could reduce the risk of opening arachnoid layer with subsequent CSF flow [16]. For this reason, it was still unclear whether ER or IR could decrease complications.

Next, we continued to focus on the complications between the two groups, but no significant difference was indicated with respect to hormone deficiency, diabetes insipidus, intraoperative CSF or postoperative CSF leakage. Sensitive analysis was used to analyze the data from intraoperative CSF leakage because of heterogeneity. The results suggested that IR attenuated intraoperative CSF leakage after removal of Taylor’ paper. We speculated that the heterogeneity may result from the use of lumbar drainage in some patients. Thus, it was difficult to determine the actual ability of IR to prevent intraoperative CSF leakage.

## Conclusions

In summary, ER could improve the prognosis of PA to some extent, but it must be emphasized that our work has some limitations. All of the included studies were cohort study without RCTs, which would have provided the best clinical evidence. In addition, some cohorts were too small to yield definitive conclusions. Furthermore, it was very difficult to conduct subgroup analysis owing to scarce samples. Consequently, well-designed prospective, large sample size, multicenter, RCTs are still needed for further certification.

## Data Availability

All data generated or analyzed during this study are included in these published articles and their supplementary information files (Shown in References 5–11).

## Abbreviations

CI: Confidence interval
CSF: Cerebrospinal fluid
ER: Extracapsular resection
IR: Intracapsular resection
NOS: Newcastle–Ottawa Scale
PA: Pituitary adenoma
RCTs: Randomized controlled trials
RR: Risk ratio
